# Characterizing psychological dimensions in non-pathological subjects through autonomic nervous system dynamics

**DOI:** 10.3389/fncom.2015.00037

**Published:** 2015-03-25

**Authors:** Mimma Nardelli, Gaetano Valenza, Ioana A. Cristea, Claudio Gentili, Carmen Cotet, Daniel David, Antonio Lanata, Enzo P. Scilingo

**Affiliations:** ^1^Department of Information Engineering & Research Centre E. Piaggio, Faculty of Engineering, University of PisaPisa, Italy; ^2^Section of Psychology, Department of Surgical, Medical, Molecular, and Critical Area Pathology, University of PisaPisa, Italy; ^3^Department of Clinical Psychology and Pychotherapy, Babes-Bolyai UniversityCluj-Napoca, Romania

**Keywords:** psychological scales, Heart Rate Variability, InterBreath Intervals series, nonlinear analysis, multiscale entropy, multivariate multiscale entropy

## Abstract

The objective assessment of psychological traits of healthy subjects and psychiatric patients has been growing interest in clinical and bioengineering research fields during the last decade. Several experimental evidences strongly suggest that a link between Autonomic Nervous System (ANS) dynamics and specific dimensions such as anxiety, social phobia, stress, and emotional regulation might exist. Nevertheless, an extensive investigation on a wide range of psycho-cognitive scales and ANS non-invasive markers gathered from standard and non-linear analysis still needs to be addressed. In this study, we analyzed the discerning and correlation capabilities of a comprehensive set of ANS features and psycho-cognitive scales in 29 non-pathological subjects monitored during resting conditions. In particular, the state of the art of standard and non-linear analysis was performed on Heart Rate Variability, InterBreath Interval series, and InterBeat Respiration series, which were considered as monovariate and multivariate measurements. Experimental results show that each ANS feature is linked to specific psychological traits. Moreover, non-linear analysis outperforms the psychological assessment with respect to standard analysis. Considering that the current clinical practice relies only on subjective scores from interviews and questionnaires, this study provides objective tools for the assessment of psychological dimensions.

## 1. Introduction

Psychological assessment refers to the practice of standardized evaluation of performance or impairment in different domains of thinking, learning and behavior. Accordingly, such an assessment can be used to characterize and quantify different behaviors in healthy subjects or to reveal the presence of behavioral disorders such as anxiety and social phobia. Depending on the factors under observation, psychological assessment can be achieved via different routes: behavioral tasks, questionnaires, or interviews. The evaluation is done by a professional (i.e., certified psychologist) in order to obtain a standardized and quantifiable information of the subject under study (Cohen et al., 1992). These approaches are useful in performing an individual assessment for which the performance of one person can be interpreted through pre-existing norms, as well as in group assessment which allows for different comparisons (within a single group or between groups) (Kenny et al., 2008). It is worthwhile noting that self-report questionnaires and interviews currently represent the standard clinical practice in diagnosing psychiatric disorders (Cohen et al., 1992; Valenza et al., 2013a, 2014c).

Nevertheless, several issues in these kinds of approaches still need to be addressed. First, the scores are obtained with subjective procedures which might be biased by possible social desirability thoughts of the subject and possible recent emotional events. Moreover, professionals need to choose the appropriate test for each psychological dimension and subject, and verify that it has good psychometric properties in order to adhere to the evidence-based paradigm (i.e., reliability and validity) (Groth-Marnat, 2003; Hunsley and Mash, 2010). To overcome these problems, several efforts have been made in psycho-physiological and bioengineering research fields to objectify the psychological assessment. In particular, physiological correlates of the central and autonomic nervous systems (CNS and ANS, respectively) have been extensively studied and taken into account (Taillard et al., 1990, 1993; Carney et al., 1995; Glassman, 1998; Stampfer, 1998; Iverson et al., 2002, 2005; Watkins et al., 2002; Calvo and D'Mello, 2010; Lin et al., 2010; Petrantonakis and Hadjileontiadis, 2011; Valenza et al., 2012a,b, 2013a,b, 2014c).

To give some significant examples, physiological correlates of mood disorders such as bipolar disorders have been found on sleep (Stampfer, 1998; Iverson et al., 2002, 2005), hormonal system (Carney et al., 1995; Glassman, 1998; Watkins et al., 2002), and ANS dynamics through heartbeat and respiratory dynamics (Taillard et al., 1990, 1993; Valenza et al., 2013a, 2014c). Moreover, as the psychological dimensions can be related to variations of emotional states, several computational methods for automatic emotion recognition have been developed using electroencephalogram (EEG) and ANS signal analysis (Taillard et al., 1990, 1993; Calvo and D'Mello, 2010; Lin et al., 2010; Petrantonakis and Hadjileontiadis, 2011; Valenza et al., 2012a,b, 2013a,b,2014c).

Here we focus on the link between ANS dynamics and psychological dimensions. This choice is justified by the fact that ANS dynamics cannot be straightforwardly changed by the subject intention and is under direct control of CNS pathways such as the prefrontal cortex, amygdala, and brainstem (Ruiz-Padial et al., 2011). Of note, dysfunctions on these CNS recruitment circuits lead to pathological effects (Heller et al., 2009) such as anhedonia, i.e., the loss of pleasure or interest in previously rewarding stimuli, which is a core feature of major depression and other serious mood disorders. Moreover, ANS monitoring is widely available, cost-effective, and can be easily performed through wearable systems such as sensorized t-shirts (Valenza et al., 2008, 2014c) or gloves (Lanatà et al., 2012), and its dynamics is thought to be less sensitive to artifact events than in the EEG case.

ANS dynamics has been demonstrated to provide effective markers of typical psychological processes. As a matter of fact, previous studies (Freeman and Nixon, 1985; Yeragani et al., 1999; Virtanen et al., 2003; Cohen and Benjamin, 2006; Shinba et al., 2008; Licht et al., 2009; Thayer et al., 2010; 2012) suggest that patients with anxiety are at increased risk for heart disease (e.g., the association between phobic anxiety or panic disorder and somatic morbidity as coronary heart disease, coronary spasm and ventricular arrhythmia). ANS markers of anxiety and panic disorders can be found through the analysis of the Heart Rate Variability (HRV), revealing an increased heart rate and decreased power in low-frequency (LF) and high frequency (HF) bands. A decreased HF spectral power of HRV was also found in patients affected by generalized anxiety disorder (Thayer et al., 1996), whereas a decreased heart rate was also found in autism spectrum disorders (Jansen et al., 2006) in response to stress. This change could be related to abnormal high basal (nor)epinephrine levels. On the contrary, increased mean heart rate associated to a reduced variability has been observed in depressed patients (Carney et al., 2005). Moreover, it has been shown how subjects reporting excessive and persistent fear of social situations are characterized by atypical ANS dynamics which is evident in variables as HRV mean, respiration rate, tidal volume, and blood pressure (Grossman et al., 2001). ANS markers gathered from non-linear analysis were related to phycological dimensions as anxiety (Cohen and Benjamin, 2006) and panic disorder through symbolic analysis (Yeragani et al., 2000). Despite the elevated number of previous studies, none of these researches have reached an acceptable level of accuracy to effectively, reliably, and objectively characterize the psychological dimensions of healthy subjects and psychiatric patients, and to forecast a clinical course. A possible reason can be related to the limited amount of ANS features and specific psychological traits that were taken into account.

Therefore, here we present a detailed study on psychological assessments through an extensive analysis of the ANS dynamics. Psychological dimensions were quantified by means of the 6 psycho-cognitive scales (see details on the series definition, estimation, and parameter extraction in Section 2.3).

In order to perform a comprehensive study, the ANS non-linear dynamics has to be taken into account. Although the detailed physiology behind such complex dynamics has not been completely clarified, it is worthwhile noting that ANS non-linear dynamics plays a crucial role in most of the underlying biological processes, as they have been proven to be of prognostic value in aging and diseases, showing robust and effective discerning and characterizing properties (Poon and Merrill, 1997; Glass, 2001; Goldberger et al., 2002; Stiedl and Meyer, 2003; Tulppo et al., 2005; Atyabi et al., 2006; Glass, 2009; Wu et al., 2009; Citi et al., 2012; Valenza et al., 2014a). Indeed, physiological systems are intrinsically non-linear systems characterized by multi-feedback interactions associated to long-range correlations (Marmarelis, 2004), likely due to the enormous amount of structural units inside them and to the various non-linear neural interactions and integrations occurring at the neuron and receptor levels. The study of the complexity of physiological signals, in particular, has led to important results in recent decades in understanding the mechanisms underlying mental illness (Yang and Tsai, 2012). Several measures of complexity have also been proposed and applied to the study of mental illness based on various biomedical signals, from EEG (Hu et al., 2006; Takahashi et al., 2010; Gao et al., 2011), to MEG (Fernandez et al., 2010), through HRV (Mujica-Parodi et al., 2005; Hu et al., 2009, 2010; Gao et al., 2013; Valenza et al., 2014b). Accordingly, in this study we investigate the role of ANS non-linear dynamics in performing the psychological assessment, with respect to the standard analysis, i.e., analysis in the time and frequency domain.

## 2. Materials and methods

### 2.1. Subjects recruitment, experimental protocol, and acquisition set-up

A group of 29 non-pathological subjects (5 males), i.e., not suffering from both cardiovascular and evident mental pathologies, was recruited to participate in the experiment. Subjects were students recruited from the Babes-Bolyai University, via an online screening questionnaire assessing their intention to take part in the study. Participation was voluntary and each subjects signed a written informed consent after the study procedure had been explained. No compensation for participation was offered. Subjects underwent a medical screening interview to assess the presence of any medical condition or medication that might have interfered with their cardiovascular data. Their age ranged from 21 to 35 and were naive to the purpose of the experiment. The group was as heterogeneous as possible in order to have a wide range of psycho-cognitive-behavioral dimensions. The experimental protocol was structured in the following two phases: (1) submission of self report psycho-behavioral tests; (2) recording of the physiological signs. More in detail, all participants were screened by 6 self-report questionnaires (see details below), which were comprised of a total of 25 sub-scales. Then, physiological signals such as ElectroCardioGram (ECG), Respiration (RSP) were simultaneously acquired during resting state condition for 25 min through the BIOPAC MP150 device. The sampling rate was 1000 Hz for all signals. We used the ECG100C Electrocardiogram Amplifier from BIOPAC inc., connected with pregelled Ag/AgCl electrodes placed following Einthoven triangle configuration. The dedicated module of BIOPAC MP150 used to record the respiration activity is RSP100C Respiration Amplifier with the TSD201 sensor, which is a piezo-resistive sensor with the output resistance within the range 5–125 KOhm and bandwidth of 0.05–10 Hz. This piezoresistive sensor changed its electrical resistance if stretched or shortened, and it was sensitive to the thoracic circumference variations occurring during respiration.

The ECG signal was used to extract the HRV series, which refer to the variation of the time intervals between consecutive heartbeats identified with R-waves (RR intervals). Two different time series were extracted from the respiration activity: InterBreath Interval time series (IBI) and InterBeat Respiration (IBR). The IBI series was obtained detecting the local maxima of each respiratory act, whereas IBR consists of the amplitude of the respiration activity signal when sampled at the R-peak times.

### 2.2. Scales for the assessment of psychological dimensions

In this work, we used a total of 6 self-report questionnaires in which, for most of them, different sub-scales are considered. The total number of sub-scales used in this experiment was 25. A Cronbach's α measure (Bland and Altman, 1997) is assigned to each scale and represents the consistency of the test. Such an α index depends on the number and average inter-correlation among the test questions. Details on each scale and related sub-scales are as follows:

- Positive and Negative Affect Schedule (PANAS, Watson et al., 1988). The PANAS contains 2 sub-scales—positive affect (PA) and negative affect (NA)—of 10 items describing emotions each. The scale has good reliability (Cronbach's α = 0.88 for the PA and 0.87 for the NA sub-scale, respectively) and good construct validity. Cronbach's α for this sample was 0.87 for PA and 0.90 for NA, supporting good internal consistency.
- Liebowitz Social Anxiety Scale (LSAS, Liebowitz, 1987). The LSAS is a self-assessment social phobia questionnaire containing 24 items describing actions done in social situations, grouped at first in 2 sub-scales (social interaction and performance). Subjects rate these situations in terms of fear/anxiety and avoidance, allowing for a total of 4 separate sub-scales. The scale presents a very good internal consistency (Cronbach's α = 0.96) as well as good convergent and divergent validity (Heimberg et al., 1999). Cronbach's α for this sample was 0.92, showing a very good internal consistency.
- Difficulties in Emotion Regulation (DERS, Gratz and Roemer, 2004). The DERS is a 36-item self-report scale measuring emotion dysregulation. The scale offers an overall score as well as scores for each of the 6 sub-scales related to DERS (Non-acceptance of Emotional Responses, Difficulties Engaging in Goal-Directed Behavior, Impulse Control Difficulties, Lack of Emotional Awareness, Limited Access to Emotion Regulation Strategies, and Lack of Emotional Clarity). Internal consistency for this scale is excellent (Cronbach's α = 0.93) and construct and predictive validity are considered adequate.
- Interpersonal Reactivity Index (IRI, Davis, 1980). The IRI is a 28-item questionnaire measuring empathy. The scale provides scores for 4 sub-scales (Fantasy, Perspective-taking, Empathic Concern, and Personal Distress), as well as a general score of empathy. Internal consistency of the four sub-scales is acceptable (ranging from α = 0.70–0.78).
- Behavioral Inhibition/Behavioral activation Scales (BIS/BAS, Carver and White, 1994). The BIS/BAS scale is composed of 20 items comprised in 4 sub-scales (Inhibition, Reward Responsiveness, Drive, and Fun Seeking), measuring behavioral inhibition and activation sensitivity. The scale has been adapted on the Romanian population, showing good construct validity and acceptable internal consistency (ranging from α = 0.62–0.81) (Sava and Sperneac, 2006).
- Zuckerman Kuhlman Personality Questionnaire (ZKPQ, Zuckerman et al., 1993). The ZKPQ represents a five-factor (Impulsive Sensation Seeking, Neuroticism-Anxiety, Aggresion-Hostility, Sociability, and Activity) personality inventory containing 99 true-false items, therefore we used 5 sub-scales. The Romanian adaptation of this scale presents adequate internal consistency (α ranging from 0.69 to 0.88) and good convergent validity (Sârbescu and Neguţ, 2012).

### 2.3. Methodology of signal processing

In this section, the methodology of signal processing applied to the Heart Rate Variability (HRV), InterBreath Interval (IBI), and InterBeat Respiration (IBR) series is reported in detail. HRV refers to the variability of the series comprised of the distances between two consecutive R-waves detected from the Electrocardiogram, i.e., the R-R intervals. IBI is the series comprised of the distances between two consecutive local maxima of the respiration activity (the two maxima within two respiratory acts), whereas IBR series is the respiratory activity sampled at times corresponding to the R-peaks. Standard and non-linear monovariate and multivariate measure are extracted from each series in order to investigate a wide set of parameters characterizing the ANS linear and non-linear dynamics acting of the cardio-respiratory control.

#### 2.3.1. Standard measures

Standard analysis was performed on HRV series in order to extract parameters defined in the time and frequency domain (Camm et al., 1996; Acharya et al., 2006; Valenza et al., 2012b). Time domain features include statistical parameters and morphological indexes. More specifically, concerning the time domain analysis, in addition to the first (meanRR) and second order moment (SDNN) of the RR intervals, so-called normal-to-normal (NN) intervals, the square root of the mean of the sum of the squares of differences between subsequent NN intervals ($RMSSD=\sqrt{\frac{1}{N-1}\sum_{j=1}^{N-1}(RR_{j+1}-RR_{j}^{2}}$) and the number of successive differences of intervals which differ by more than 50 ms, expressed as a percentage of the total number of heartbeats analyzed ($pNN50=\frac{NN50}{N-1}100\%$) were calculated. Moreover, the triangular index (TINN) was estimated as the base of a triangle which better approximated the NN interval distribution (the minimum square difference is used to find such a triangle).

Concerning the frequency domain analysis, several features were calculated from the Power Spectral Density (PSD) analysis. In this work, PSD was estimated by using the Welch's periodogram, which uses the FFT (Fast Fourier Transform) algorithm. Window's width and overlap were chosen as a best compromise between the frequency resolution and variance of the estimated spectrum. Given the PSD, three spectral bands are defined as follows: VLF (very low frequency) with spectral components below 0.04 Hz; LF (low frequency), ranging between 0.04 and 0.15 Hz; HF (high frequency), comprising frequencies between 0.15 and 0.4 Hz. For each of the three frequency bands, the frequency having maximum magnitude (VLF peak, LF peak, and HF peak), the power expressed as percentage of the total power (VLF power %, LF power %, and HF power %), and the power normalized to the sum of the LF and HF power (LF power nu and HF power nu) were also evaluated. Moreover, the LF/HF power ratio was calculated.

#### 2.3.2. Non-linear analysis

From the HRV, IBI, and IBR series, several non-linear measures were calculated. Such indices refer to the estimation and characterization of the phase space (or state space) of the physiological system generating the series. The phase space estimation involved the Takens method (Takens, 1981; Casdagli et al., 1991) and three parameters: *m*, the embedding dimension, which is a positive integer, τ, the time delay, and *r*, which is a positive real number and represents the margin of tolerance of the trajectories within the space. Takens theory allows for the reconstruction of the dynamic systems of different nature from time series through the method of “delayed outputs.” Starting from a time series

X=[u(T),u(2T),…u(NT)]

the attractors of the discrete dynamical system are rebuilt in a *m*-dimensional space, operating a delay τ on the signal. This allows achieving *N* − (*m* − 1) signals of length *m* starting from only one:

$$\left\{ \begin{array}{l} X_{1}=[u(T),u(2T),\ldots u(mT)]\\ X_{2}=[u(2T),u(2T+2\tau ),\ldots u(2T+(m-1)\tau )]\\ \ldots \\ X_{N-(m-1)}=[u(N-(m-1))T),\ldots u(N-(m-1))T\\ \;+(m-1)\tau )] \end{array}\right.$$

The various vectors *X_j_* are the “delayed coordinates” and the derived m-dimensional space is called “reconstructed space.” From the state space theory, several ANS non-linear parameters can be derived using the following analyses:

- Poincarí Plot
- Recurrence Plot
- Correlation dimension, Approximate, and Sample Entropy
- Detrended Fluctuation Analysis
- Multiscale Entropy and Multivariate Multiscale Entropy Analysis

##### 2.3.2.1. Poincarí Plot

This technique quantifies the fluctuations of the dynamics of the time series through a map of each point RR(n) of the RR series vs. the previous one. The quantitative analysis from the graph can be made by calculating the standard deviation of the points by the straight line *RR*_*j* + 1_ = *RR_j_*. The first standard deviation, SD1, is related to the points that are perpendicular to the line-of-identity and describes the short-term variability, whereas the second, SD2, describes the long-term variability.

##### 2.3.2.2. Recurrence Plot

RP is a graphical method to investigate and quantify the time series complexity. The estimation starts from vectors

$$\begin{array}{l} u_{j}=(RR_{j},RR_{j+\tau },\ldots ,RR_{j+(m-1)\tau })\\ \;j=1,2,\ldots ,N-(m-1)\tau . \end{array}$$

RP is a symmetrical square matrix of zeros and ones, whose dimensions are *N* − (*m* − 1)τ, and each element is given by

$$RP(j,k)=\;\{\begin{array}{ll} 1 & \;if\;d(u_{j}-u_{k}\leq r)\\ 0 & \text{otherwise} \end{array}$$

where *d* is the Euclidean distance.

Several features can be extracted from the RP by means of the Recurrence Quantification Analysis (RQA). In particular, in this study the following RQA indices were taken into account: longest diagonal line (RP Lmax) and average diagonal line length (RP Lmean), divergence (RP DIV), the percentage of recurrence points which form diagonal lines recurrence rate, determinism (RP DET), trend (RP REC) and entropy (RP ShanEn) (Zbilut et al., 1990; Marwan et al., 2002, 2007).

##### 2.3.2.3. Correlation dimension, Approximate, and Sample Entropy Measures

Starting from the vectors *X*_1_, *X*_2_, …, *X*_*N* − *m* + 1_ in ???^*m*^, the distance between two vectors *X_i_* and *X_j_*, according to the definition of Takens applied to high dimensional deterministic systems is given by Takens (1981) and Schouten et al. (1994):

(1) $$d\left[X_{i},X_{j}\right]=max_{k=1,2,\ldots ,m}\left|u\left(i+k-1\right)-u\left(j+k-1\right)\right|$$

For each *i*, with 1 ≤ *i* ≤ *N* − *m* + 1, we measured a parameter *C^m^_i_*(*r*):

(2) $$\begin{array}{l} C_{i}^{m}(r)=\frac{\text{Number of j such that}(d[X_{i},X_{j}]\leq r)}{N-m+1} \end{array}$$

and we defined

(3) $$\begin{array}{l} C^{m}(r)=\frac{\sum_{i=1}^{N-m+1}\log C_{i}^{m}(r)}{N-m+1} \end{array}$$

The correlation dimension (CD) is given by Theiler (1987)

$$CD=lim_{r\to 0}lim_{N\to \inf}\frac{logC^{m}(r)}{logr}$$

The calculation of ApEn used in this study refers to the expression (Pincus, 1991; Fusheng et al., 2001):

(4) $$\begin{array}{l} \text{ApEn}(\text{m},\text{r},\text{N})=[C^{m}(r)-C^{m+1}(r)] \end{array}$$

*SampEn* is a remake of *ApEn* and measures the number of pairs of vectors of length *m* considered “neighbors,” i.e., whose distance is less than *r*, even if the dimension of pattern increases from *m* to *m* + 1. Unlike *ApEn*(*m*, *r*, *N*), *SampEn* does not include the distance of vectors with themselves, i.e., self-matches, as suggested in the later work of Grassberger and co-workers (Grassberger and Procaccia, 1983; Grassberger, 1988) and it has the advantage of being less dependent on time series length, showing relative consistency over a broader range of possible *r*-, *m*-, and *N*-values. By renaming *C^m^*(*r*) parameters without self-matches with the notation *U^m^*(*r*), *SampEn* is calculated by the following expression (Richman and Moorman, 2000):

(5) $$\begin{array}{l} \text{SampEn}(\text{m},\text{r},\text{N})=-\ln\frac{U^{m+1}}{U^{m}} \end{array}$$

##### 2.3.2.4. Detrended Fluctuation Analysis

The detrended fluctuation analysis features (DFA1 and DFA2) (Peng et al., 1995; Penzel et al., 2003) were evaluated to study short- and long-term autocorrelation of the HRV series. The algorithm foresaw the estimation of the series

$$y(k)=\sum_{j=1}^{k}\left(RR_{j}-\bar{RR}\right)$$

*k* = 1, …, *N*. This series was divided into segments of equal length *n* and for each segment the linear approximation (least square fit, *y_n_*) was computed. Then root-mean-square fluctuation was calculated

$$F(n)=\sqrt{\frac{1}{N}\overset{N}{\underset{k=1}{\sum }}{\left(y(k)-y_{n}(k)\right)}^{2}}$$

Making a double log graph between *log(F(n))* and different values of *n*, the slope of the regression line is the α scaling exponent. DFA1 and DFA2 features represent this slope between the ranges 4 ≤ *n* ≤ 16 and 16 ≤ *n* ≤ 64.

##### 2.3.2.5. Multiscale Entropy and Multivariate Multiscale Entropy Analysis

Multiscale Entropy Analysis (MSE) is a powerful methodology based on the SampEn estimation. MSE was applied in several fields such as study of human gait dynamics (Costa et al., 2003), enhancement of postural complexity (Costa et al., 2007), and synthetic RR time series (Costa et al., 2002). MSE can be an effective non-linear method to collect information about physiological systems whose dynamics is associated to multiple different scales. This method is based on the application of sample entropy method to course-grained time series constructed from a one-dimensional discrete time series by averaging the data points within non-overlapping windows of increasing length, σ. Given a time series {*x*_1_, …, *x_i_*, …, *x_N_*} and a scale factor σ, each element of a course-grained series $\left\{y^{\left(\sigma \right)}\right\}$ is calculated using the equation

(6) $$\begin{array}{l} y_{j}^{\left(\sigma \right)}=\frac{1}{\sigma }\sum_{i=(j-1)\sigma +1}^{j\sigma }x_{i},1\leq j\leq N/\sigma \end{array}$$

The length of each coarse-grained time series is equal to the length of the original time series divided by σ. The second step consists in the computation of *S*ampEn (Richman and Moorman, 2000; Lake et al., 2002) algorithm on these series. Previous studies in which MSE algorithm was applied to physiological data use the standard value *m* = 2 for the pattern dimension (Costa et al., 2003; Leistedt et al., 2011). In this work the choice of the right *r* was performed by a method already used in the liter SampEn values were calculated for scale factors σ which were in a range from 1 to 20 and the same process was carried out on HRV, IBI, and IBR series. The complexity index (CI) was measured as the area under the curve of MSE graph and it can be calculated for short time scales, from 1 to 8 (short CI), and for higher time scales, up to 20 (long CI) (Leistedt et al., 2011).

Besides MSE analysis, we performed the Multivariate Multiscale Entropy (MMSE) (Ahmed and Mandic, 2011, 2012) analysis. This algorithm allows performing MSE analysis using multivariate time series. In this work, MMSE was used to quantify the complexity of the series derived from the electrocardiogram and breath. In particular, MMSE results were obtained on the bivariate series HRV-IBI, and HRV-IBR through the estimation of the CI indices (as described above on MSE). Before the MMSE calculation, the involved time series are scaled in the range between 0 and 1 to prevent that the different amplitudes may influence the complexity complexity (Ahmed and Mandic, 2011).

##### 2.3.2.6. Symbolic Analysis

Symbolic analysis (Yeragani et al., 2000; Porta et al., 2001; Baumert et al., 2002; Bella and Montano, 2005; Tobaldini et al., 2009; Caminal et al., 2010) is another powerful non-linear method which was applied on HRV data series. For each HRV series gathered from each subject, 6 levels were constructed evenly dividing the amplitude range of the samples, and a symbol (from 0 to 5) was assigned to each data sample according to the level of belonging. Then, a window of three consecutive points moves along the HRV series, and three possible configurations are identified when running all the signal: the three points belong to the same level, i.e., no variation (0V), two consecutive points belong to the same level and one to another, i.e., one variation (1V), and the remaining cases, i.e., two variations (2V). The number of patterns falling into each group (0V, 1V, 2V) and the percentage of the total (0V%, 1V%, 2V%) were calculated and used as features. Previous studies support the hypothesis that an increase of 0V patterns is related to an activation of the sympathetic activity, an increase of 2V patterns is related to an increase of the parasympathetic activity, and increases of 1V patterns is associated to a simultaneous increase of both parasympathetic and sympathetic activities.

## 3. Experimental results

Experimental results are expressed in terms of statistical and correlation analysis. In the literature it can be found the threshold score of each questionnaire above which the behavior of the subject results to show altered psycho-cognitive-behavioral traits. Among all the sub-scales we only considered those where the subjects spread out over a wide range of scores in order to identify two groups, one below and the other above the threshold. For each scale we identified two groups of subjects separated by the median. In order to have two groups numerically equivalent, we selected and investigated only these scales where the median was congruent with the threshold reported in the literature. In addition, for each of the 16 scales we verified that maximum and minimum scores of each group were in the tails of the population distribution reported by the literature. In other words, for each psychological subscale, the median value of the subjects score is calculated to identify two groups: one comprised of the subjects having scores below the median, and one comprised of the subjects having scores above the median. Only 16 out of 25 sub-scales divided the subjects in two groups numerically comparable, therefore we performed the statistical analysis on the scores obtained in these 16 sub-scales. The reference values from the literature about these sub-scales are evaluated on the control groups used in several previous works. For example we considered a sample of 103 subjects (age = 27.00 ± 8.80) for IRI Empathic Concern and IRI Personal Distress sub-scales, referring to a study which explored the relationship among psychological mindedness and several aspects of awareness which comprended this indices of empathy (Beitel et al., 2005) and a sample of 582 subjects for IRI fantasy sub-scale taking this data from a guide study on the empathy scales (Davis, 1980). For the two PANAS sub-scales, a group of 537 volunteers aged 18–91 was in a work that tried to evaluate the reliability and validity of the PANAS (Crawford and Henry, 2004), and 53 participants (age = 34.32 ± 10.50) were asked to answer to the LSAS questionnaires to demonstrate that this method may be employed in the assessment of social anxiety disorder (Fresco et al., 2001; Rytwinski et al., 2009). As a reference for the values of BIS and BAS sub-scales we chose a previous study where the answers of of 2725 individuals aged 18–79 were observed to validate the application of this scale to measure the behavioral inhibition and activation and its correlation with depression and anxiety (Jorm et al., 1998). The threshold value of the answers of a group of 639 participants in a study of the shortened form of the questionnaire, was taken in account for ZKPQ Impulsive Sensation Seeking and Activity sub-scales (age = 22.31 ± 5.08) (Aluja et al., 2003). At last, as regards DERS subscales, a study on 260 subjects in order to explore the factor structure and psychometric properties of DERS measures (age = 23.10 ± 5.67) was used as reference for DERS Awareness (Gratz and Roemer, 2004) and a reference sample of 42 individuals (age = 24.24 ± 4.38) was considered for the other DERS sub-scales, extracted from a research which compared the values of the this psychological tests on depressed patients and healthy subjects (Ehring et al., 2008).

In the statistical analysis, for each psychological sub-scale and for each ANS feature, we applied the Mann-Whitney test in order to evaluate whether the two groups were statistically different. Moreover, the non-parametric Spearman correlation coefficient was calculated between each psychological sub-scale and ANS feature.

### 3.1. Statistical analysis

As mentioned above, for each ANS feature, Mann-Whitney non-parametric *U*-tests were used to test the null hypothesis of having no statistical difference between two groups. The use of such a non-parametric test is justified by having non-gaussian distribution of the samples (*p* < 0.05 of the null hypothesis of having gaussian samples of the Kolmogorov-Smirnov test).

**Figure 1:**
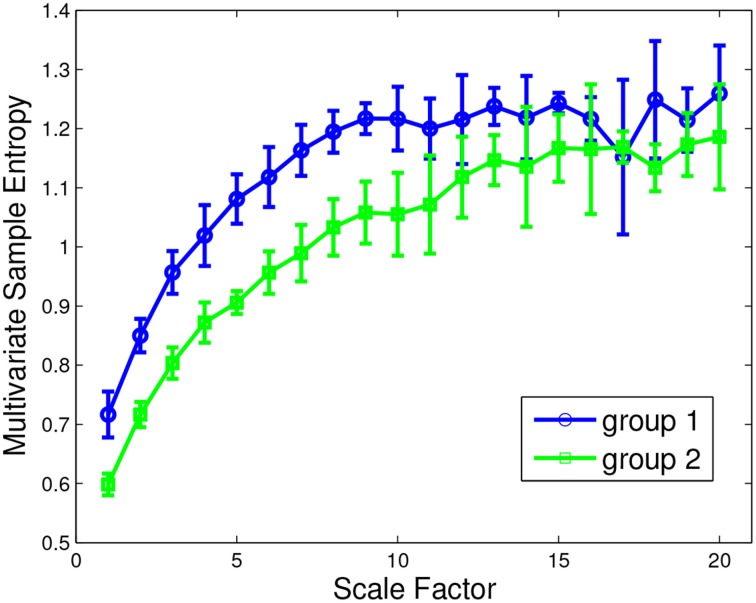
**Exemplary plot of Multivariate Multiscale Entropy analysis applied to HRV-IBI series in discerning the two groups (under the median-lower scores: group 1; over the median-higher scores: group 2) according to scores gathered from the DERS Non-Accept sub-scale**.

Concerning features from HRV standard analysis, 8 sub-scales (LSAS Anxiety of Performance, DERS Non-Acceptance, DERS Awareness, IRI fantasy, IRI Empathic Concern, ZKPQ Activity, ZKPQ Impulsive Seeking Sensation, BAS) showed significant discerning capability mostly through frequency domain parameters (see details in Table 1). Concerning ANS features coming from non-linear analysis, 9 sub-scales (PANAS Positive Affect, DERS non-Acceptance, DERS Impulse, DERS Awareness, DERS Strategies, IRI Empathic Concern, BIS, BAS, ZKPQ Activity) showed significant differences considering monovariate and multivariate measures (see details in Table 2). An exemplary plot showing the discerning capability of MMSE analysis on DERS Non-Accept sub-scale is shown in Figure 1.

**Table 1:** **Statistical results related to standard HRV features (*U*-test)**.

**Scales**	**Sub-scales**	**Statisticalresults**	
		**Features**	***p*-value**
LSAS	LSAS Anx P	↓ VLF peak	<0.05
		↓ HF peak	<0.03
DERS	DERS Non-Accept	↓ LF peak	<0.03
		↑ VLF power	<0.05
	DERS Awareness	↓ TINN	<0.05
		↑ LF power nu	<0.05
		↓ HF power	<0.03
		↓ HF power %	<0.01
		↓ HF power nu	<0.05
		↑ LF/HF	<0.05
IRI	IRI fantasy	↓ VLF power	<0.05
		↑ HF power %	<0.05
	IRI EC	↑ RMSSD	<0.01
		↑ Pnn50	<0.01
		↓ LF power nu	<0.03
		↑ HF power	<0.01
		↑ HF power %	<0.03
		↑ HF power nu	<0.03
		↓ LF/HF	<0.03
BIS/BAS	BAS	↓ LF power %	<0.01
ZKPQ	ZKPQ Impuls.S.S.	↓ LF power %	<0.03
	ZKPQ Activity	↓ LF power nu	<0.03
		↑ HF power nu	<0.03
		↓ LF/HF	<0.03

*VLF, Very Low Frequency; LF, Low Frequency; HF, High Frequency; nu, normalized units; TINN, width of triangular approximation to NN-interval frequency distribution; RMSSD, square root of mean squared forward differences of successive NN intervals; Pnn50, proportion of successive NN interval differences>50 ms ↑ indicates that an increase of the test score is associated to an increase of the feature value. ↓ indicates that an increase of the test score is associated to a decrease of the feature value*.

**Table 2:** **Statistical results related to non-linear features (*U*-test)**.

**Scales**	**Sub-scales**	**Statistical results**	
		**Features**	***p*-value**
PANAS	PANAS PA	↓ MSE IBI (long CI)	<0.05
DERS	DERS non-Accept	↓ MSE IBR (short CI)	<0.05
		↓ MSE IBR (longCI)	<0.05
		↓ MMSE HRV-IBI (shortCI)	<0.01
		↓ MMSE HRV-IBI (longCI)	<0.01
	DERS Impulse	↓ 2V	<0.05
	DERS Awareness	↓ MMSE HRV-IBR (longCI)	<0.03
		↓ MMSE HRV-IBR (shortCI)	<0.05
	DERS Strategies	↓ 2V%	<0.05
IRI	IRI EC	↑ CD	<0.01
		↓ SD1	<0.01
		↓ DFA1	<0.05
		↓ MMSE HRV-IBR (shortCI)	<0.03
		↓ MMSE HRV-IBR (longCI)	<0.03
		↑ 1V%	<0.05
BIS/BAS	BIS	↑ CD	<0.03
	BAS	↓ MMSE HRV-IBR (shortCI)	<0.03
		↓ 0V	<0.03
		↓ 0V%	<0.03
ZKPQ	ZKPQ_Activity	↑ ApEn	<0.01
		↑ SampEn	<0.01
		↑ RP Lmax	<0.05

*MSE HRV, Multiscale Entropy on HRV series; MSE IBR, Multiscale Entropy on IBR series; MSE IBI, Multiscale Entropy on IBI series; MMSE HRV-IBR, Multivariate Multiscale Entropy on bivariate HRV and IBR series; MMSE HRV-IBI, Multivariate Multiscale Entropy on bivariate HRV and IBI series; CI, Complexity Index. ApEn, Approximate Entropy, SampEn; Sample Entropy, 0V, number of patterns with none variation in the amplitude; 0V%, 1V%, 2V%, percentage of the total patterns with zero, one or two variations in the amplitude; SD1, Standard Deviation of PoincarÃl' Plot related to the points that are perpendicular to the line-of-identity; DFA1, Detrended Fluctuation Analysis (first slope); RP Lmax, Recurrence Plot (longest diagonal line); CD, Correlation Dimension ↑ indicates that an increase of the test score is associated to an increase of the feature value. ↓ indicates that an increase of the test score is associated to a decrease of the feature value*.

**Figure 2:**
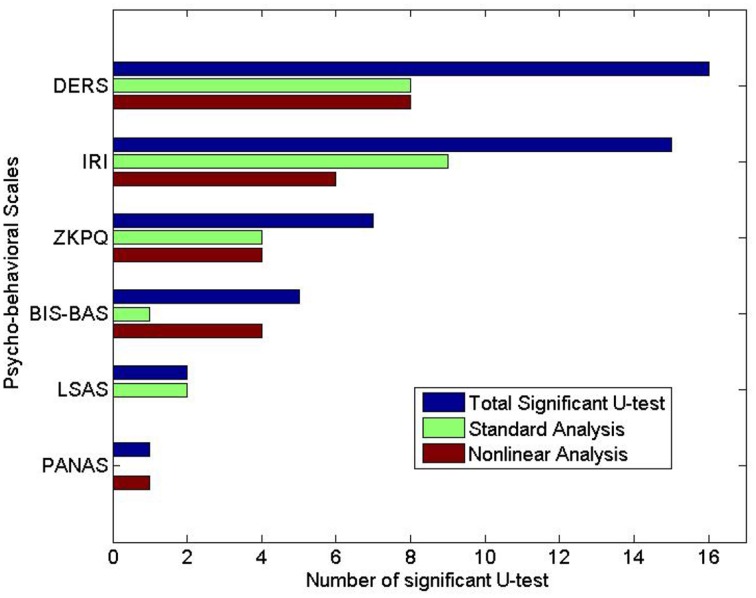
**Number of features with significant *p*-values (*p* < 0.05) given by the Mann-Whitney tests**. For each phycological scale, the number of significant parameter of standard analysis, non-linear analysis, and the total are shown.

To summarize the results, all the extracted features were able to discern the two groups in 12 out of 16 sub-scales. More specifically, standard HRV analysis provided exclusive information, i.e., not overlapped with that coming from the non-linear analysis, on the psychological assessment in only 2 sub-scales, whereas features from ANS non-linear dynamics exclusively discriminated the two groups in 4 sub-scales (see details in Figure 2).

### 3.2. Correlation analysis

The Spearman correlation coefficient was used to show the relationship between the values of each features through all the subjects and the relative score for each sub-scale. Accordingly, the coefficient ρ and *p*−value expressing the probability that no correlation between the two variables exist, were assigned for each sub-scale and each feature. Results are shown in Tables 3, 4.

**Table 3:** **Spearman correlation test results related to standard HRV features**.

**Sub-scales**	**Features**	**Spearman test results**	
		**rho**	***p*-value**
DERS Awareness	LF power nu	0.41	<0.03
	HF power	−0.41	<0.03
	HF power %	−0.49	<0.01
	HF power nu	−0.43	<0.03
	LF/HF	0.42	<0.03
IRI EC	Pnn50	0.43	<0.03
BAS	LF power %	−0.52	<0.01
ZKPQ Impuls.S.S.	LF power	−0.39	<0.05
	LF power %	0.52	<0.01
ZKPQ Activity	HF peak	0.41	<0.03
	LF power nu	−0.48	<0.01
	HF power %	0.44	<0.03
	HF power nu	0.48	<0.01
	LF/HF	−0.48	<0.01

*VLF, Very Low Frequency; LF, Low Frequency; HF, High Frequency, nu, normalized units; TINN, width of triangular approximation to NN-interval frequency distribution; RMSSD, square root of mean squared forward differences of successive NN intervals; Pnn50, proportion of successive NN interval differences > 50 ms*.

**Table 4:** **Spearman correlation test results related to non-linear HRV, IBI, IBR features**.

**Sub-scales**	**Features**	**Spearman test results**	
		**rho**	***p*-value**
LSAS Anx P	0V %	0.40	<0.05
DERS Non-Accept	MSE IBI (short CI)	−0.45	<0.05
	MSE IBI (long CI)	−0.55	<0.01
DERS Goals	0V	−0.37	<0.05
	0V%	−0.42	<0.03
	2V	−0.39	<0.05
	2V%	−0.37	<0.05
DERS Impulse	MSE IBR (long CI)	−0.43	<0.05
	0V	0.37	<0.05
	0V%	0.39	<0.05
DERS Awareness	DFA1	0.37	<0.05
	MSE IBR (long CI)	0.50	<0.03
DERS Strategies	0V	−0.44	<0.03
	0V%	−0.47	<0.03
	2V%	−0.41	<0.03
IRI EC	CD	0.46	<0.03
	MMSE HRV-IBR (long CI)	−0.47	<0.03
BIS	CD	0.39	<0.05
ZKPQ Impuls.S.S.	MSE HRV (short CI)	−0.44	<0.03
ZKPQ Activity	ApEn	0.48	<0.01
	SampEn	0.52	<0.01
	DFA1	−0.47	<0.01
	RP Lmax,RP DET,RP REC	−0.49	<0.01
	1V%	0.45	<0.03

*MSE HRV, Multiscale Entropy on HRV series; MSE IBR, Multiscale Entropy on IBR series; MSE IBI, Multiscale Entropy on IBI series; MMSE HRV-IBR, Multivariate Multiscale Entropy on bivariate HRV and IBR series; MMSE HRV-IBI, Multivariate Multiscale Entropy on bivariate HRV and IBI series; CI, Complexity Index. ApEn, Approximate Entropy; SampEn, Sample Entropy; 0V, number of patterns with none variation in the amplitude; 0V%, 1V%, 2V%, percentage of the total patterns with zero; one or two variations in the amplitude; SD1, Standard Deviation of PoincarÃl' Plot related to the points that are perpendicular to the line-of-identity; DFA1, Detrended Fluctuation Analysis (first slope); RP Lmax, Recurrence Plot (longest diagonal line); RP DET, Recurrence Plot (determinism); RP REC, Recurrence Plot (trend); CD, Correlation Dimension*.

We found that ANS features related to the linear HRV dynamics are significantly correlated with 5 sub-scales, reaching absolute values of ρ up to 0.52 (BAS and ZKPQ Impulsive Sensation Seeking). Moreover, 10 sub-scales are significantly correlated with markers of ANS non-linear dynamics, reaching absolute values of ρ up to 0.55 (DERS Non-Acceptance).

Although the correlation coefficient is not very high, it is, however, a very interesting result to be further validated and confirmed.

**Figure 3:**
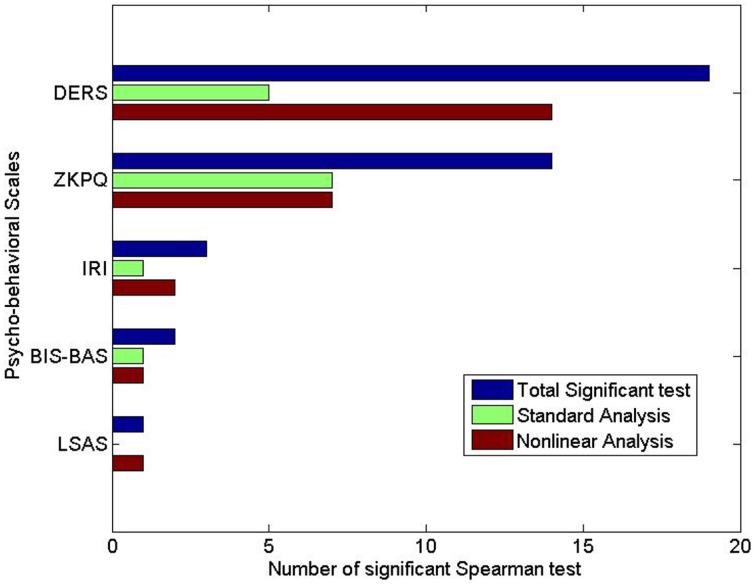
**Number of features with significant *p*-values (*p* < 0.05) given by the Spearman non-parametric correlation coefficient**. For each phycological scale, the number of significant parameter of standard analysis, non-linear analysis, and the total are shown.

The number of features with significant *p*-values (*p* < 0.05) given by such a correlation coefficient is shown in Figure 3 for each phycological dimension.

## 4. Discussion and conclusion

In conclusion, we found several ANS biomarkers of psychological dimensions in non-pathological subjects. Such biomarkers are derived from the standard and complexity analysis of ANS measures such as HRV, IBI, and IBR series. We found that dimensions related to difficulties in emotion regulation (DERS), interpersonal reactivity (IRI), behavioral activation or inhibition (BIS/BAS), sensation-seeking and activity (ZKPQ), and anxiety performance (LSAS) are always associated to changes in the HRV dynamics, quantified using time and frequency domain indices (see Table 1). As all the scale define different psychological dimensions, it is very difficulty to give a common interpretation of features through them. The LF/HF ratio decrease, associated to increased questionnaires scores, characterizes the ZKPQ activity and IRI empathic concern, whereas an opposite trend is found for the awareness of difficulties in emotion regulation (DERS). HRV time domain indices such as TINN, Pnn50, and RMSSD are effective only to characterize the empathic concern and emotion regulation. These results, gathered from statistical analyses of standard HRV parameters, are further confirmed by the correlation analyses whose details are shown in Table 3.

It is worthwhile noting that the HF power decreases with the DERS score. According to the literature (Porges, 1991, 1992), vagal tone is associated to the ability of emotional self-regulation and high flexibility and adaptability to environmental changes. According to our results, when an emotion dysregulation occurs, the sympathetic activity increases.

Other evidences supporting our results can be found in the current literature (Freeman and Nixon, 1985; Yeragani et al., 1999; Virtanen et al., 2003; Cohen and Benjamin, 2006; Shinba et al., 2008; Licht et al., 2009; Thayer et al., 2010; 2012) which suggest that patients with anxiety disorders revealed a decreased power in the HRV-LF bands.

Concerning the ANS non-linear dynamics, several biomarkers of psychological dimensions were found in complexity measures such as sample entropy, monovariate and multivatiate multiscale entropy, short- an long-term correlations, correlation dimension, recurrence and symbolic analysis in characterizing dimensions as positive and negative affect (PANAS), social phobia (Liebowitz Social Anxiety Scale, LSAS), difficulties in emotion regulation (DERS), Interpersonal reactivity (IRI), behavioral inhibition or activation (BIS/BAS), and sensation-seeking and activity (ZKPQ). Our results on non-linear ANS markers for psychological dimensions confirm the previous findings (Yeragani et al., 2000; Cohen and Benjamin, 2006) and provide a wider portrait of the complexity modulation associated with behavioral characters.

Figures 2, 3 report the number of statistically significant features given by Mann-Whitney and Spearman non-parametric correlation, respectively. It is worthy to note that the non-linear features are overall more than those extracted from standard analysis, confirming that complexity dynamics measures play a relevant role in assessing the psycho-physiological dimensions.

Finally, some prudential considerations should be made. The patterns of physiological signals are acquired in rest conditions right after performing the test and the assumption behind the experiment is that the psychological assessment acted as an affective elicitation. Results have to be considered as preliminary to future experiments where subjects experience an actual affective dimension while they are monitored. Nevertheless, it is worthwhile pointing out that complexity measures can be considered promising markers to assess the psychological traits. Is important to underline that such interest is not diminished by the difficulty in giving a physiological meaning to complexity measurements. In this sense and more in generally, we underscored how our data suggested the possibility of an ANV fingerprinting of psychological dimensions. Therefore, beyond their precise physiological meaning, our results have interesting consequences for the psychometric and clinical fields. Our approach may be promising in describing the psychological dimensions as a combination of different features, providing a full classification of psychological characteristics through a baseline ECG acquisition. However, more studies with a much higher number of subjects are needed to test the reliability and the feasibility of these potential clinical implications. Furthermore, to test if our methodology could also be extended to the extremes of the psychological dimensions, these studies should also include pathological samples (e.g., diagnosed subjects). Should that prove to be the case, this approach might hold promise as a tool for providing an external validation to psychological diagnosis.

### Conflict of interest statement

The authors declare that the research was conducted in the absence of any commercial or financial relationships that could be construed as a potential conflict of interest.
